# Dynamic Covalent Sulfur‐Selenium Rich Polymers via Inverse Vulcanization for High Refractive Index, High Transmittance, and UV Shielding Materials

**DOI:** 10.1002/marc.202400998

**Published:** 2025-01-15

**Authors:** Jinhong Jia, Yao Chai, Xingwei Xun, Yan Gao, Tongsen Qiao, Xiong Wang, Xi‐Cun Wang, Tom Hasell, Xiaofeng Wu, Zheng‐Jun Quan

**Affiliations:** ^1^ College of Chemistry and Chemical Engineering, Gansu International Scientific and Technological Cooperation Base of Water‐Retention Chemical Functional Material Northwest Normal University Lanzhou Gansu 730070 P. R. China; ^2^ Lanzhou Petrochemical Branch of China National Petroleum Corporation Lanzhou Gansu 730060 P. R. China; ^3^ Lanzhou Petrochemical Research Center PetroChina Petrochemical Research Institute Lanzhou Gansu 730060 P. R. China; ^4^ Materials Innovation Factory Department of Chemistry University of Liverpool Liverpool L69 7ZD UK

**Keywords:** adhesion, inverse vulcanisation, selenium, sulfur polymer, UV shielding

## Abstract

Recent advancements in inverse vulcanization have led to the development of sulfur‐rich polymers with diverse applications. However, progress is constrained by the harsh high‐temperature reaction conditions, limited applicability, and the generation of hazardous H_2_S gas. This study presents an induced IV method utilizing selenium octanoic acid, yielding sulfur‐selenium rich polymers with full atom economy, even at a low‐temperatures of 100–120 °C. The resultant sulfur‐selenium rich polymers exhibit exceptional optical properties: 1) A high refractive index, reaching 1.89 when the total sulfur‐selenium content is 65%; 2) Excellent UV shielding capabilities, blocking ultraviolet rays while permitting 95.1–98.6% transmission of visible light; 3) Notable transparency, with polymer films of 0.94 mm thickness exhibiting good transparency under natural light. The materials also demonstrate environmental stability under prolonged exposure to hot or cold conditions. Additionally, the polymers display adhesive strength as evidenced by two adhered glass slides with the material lifting weights of up to 20 kg without any displacement in their glued area. These properties provide a new avenue for sulfur‐selenium rich materials to be implemented in high‐precision optical instruments with unique characteristics.

## Graphical abstract

An induced inverse vulcanization method utilizing selenium octanoic acid, yielding sulfur‐selenium rich polymers with full atom economy, even at a low‐temperatures of 100‐120 °C. The resultant sulfur‐selenium rich polymers exhibit exceptional optical properties, ultra‐violet shielding capabilities, notable transparency to visible light, environmental stability under prolonged exposure to hot or cold conditions, and adhesive strength.

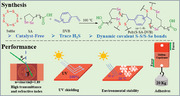

## 1 Introduction

**Figure 1:**
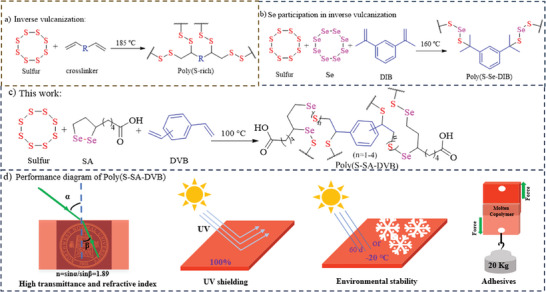
a) Tradition‐heating Inverse vulcanization. b) Se participation in inverse vulcanization. c) Preparation of sulfur‐selenium rich Polymers. d) Performance diagram of Poly(S‐SA‐DVB).

Since Pyun^[^ ^1^ ^]^ and co‐workers pioneered the concept of inverse vulcanization (IV), which involves the polymerization of elemental sulfur and unsaturated olefins to form sulfur‐rich polymers, this chemistry has garnered significant attention and found extensive applications in fields including energy storage, functional materials, and environmental remediation.^[^ ^2^ ^]^ However, the generally required high‐temperature reaction condition (>160 °C) has been problematic and hindered IV development regarding both IV chemistry itself and applications of resultant polymers. Numerous efforts have been contributing to tackle this key issue and several low‐temperature IV technologies have been developed, including metal‐catalyzed IV,^[^ ^3^ ^]^ organic alkali‐catalyzed IV,^[^ ^4^, ^5^ ^]^ ball mill IV,^[^ ^6^ ^]^ photocatalytic IV,^[^ ^7^, ^8^ ^]^ electrocatalytic IV,^[^ ^9^ ^]^ and others^[^ ^10^, ^11^ ^]^ (**Figure** **1**a). Furthermore, thioctic acid has been used as a sulfur source to prepare high‐performance sulfur‐rich polymers at lower temperature as well,^[^ ^12^, ^13^, ^14^, ^15^, ^16^, ^17^, ^18^, ^19^, ^20^ ^]^ affording unique properties for the resultant polymers such as self‐healing abilities, adhesive properties, therapeutic behavior, etc. These investigations of IV reactions at lower temperatures not only enable convenient and energy‐efficient methods to produce sulfur‐rich polymers with desired properties, but also expand the application of those resultant sulfur‐rich polymers to various new areas.

A range of salient and innate properties for specific applications of sulfur‐rich polymers has been demonstrated, such as cathode materials for lithium‐sulfur batteries,^[^ ^21^ ^]^ heavy metal adsorption,^[^ ^22^ ^]^ and optical materials. Among those, sulfur‐rich polymers with high refractive index (RI) and infrared lens/imaging properties are more distinctive. Pyun and co‐workers first exemplified RI with a value of 1.8 for sulfur‐rich polymers by reacting S_8_ with 1,3‐diisopropenylbenzene (DIB) with sulfur content ranging from 50 to 80 wt%.^[^ ^23^ ^]^ Interestingly, by replacing DIB with 1,3,5‐triisopropenylbenzene, a more tightly cross‐linked polymeric materials with transparency and a high RI(>1.75) guaranteed, possessing glass transition temperature (*T*_g_) of 100 °C.^[^ ^24^ ^]^ Meanwhile, Char and co‐workers developed a higher RI (>1.85) for a similar sulfur‐rich polymer by using divinylbenzene (DVB) as a cross‐linking agent, affording thermally stable materials (*T*_g_ >100 °C) with good transparency.^[^ ^25^ ^]^ An even higher RI of over 1.97 (632.8 nm) for pSDVB sCVD film was reported by Im and co‐workers via a new method named sulfur chemical vapor deposition (sCVD), using elemental sulfur and DVB.^[^ ^26^ ^]^ Polystyrene‐sulfur nanocomposites with a moderate RI of 1.67 was explored by Alhassan and co‐workers via introducing IV on polystyrene substrate.^[^ ^27^ ^]^ Boyd et al. achieved a high RI (>1.9) with excellent transparency for sulfur‐rich polymers generated by IV reactions using tetravinyl tin as a comonomer.^[^ ^28^ ^]^ Significant enhanced RI with a maximum value of 2.1 for sulfur‐rich polymers achieved by Pyun et al. via integrating selenium element into the IV reaction system, affording polymeric sulfur‐selenium materials^[^ ^29^ ^]^ (Figure 1b). Notably, these materials demonstrated exceptional infrared (IR) transparency as well.^[^ ^30^, ^31^, ^32^, ^33^, ^34^, ^35^ ^]^ On the other hand, materials containing germanium and silicon semiconductors, as well as chalcogenide glasses, have high refractive indices ranging from 2.0 to 4.0. However, the cost of raw materials, toxicity, and processing difficulties significantly restrict the applications of these materials.^[^ ^36^, ^37^, ^38^ ^]^ Therefore, the exploration of polymeric sulfur materials with high RI property as alternative for prominent optical materials is appealing and promising, attributing to not only the abundant and cost‐effective raw materials, but also the diverse functionality of resultant polymers. The two types of dynamic chemical bonds in selenium octanoic acid (SA): dynamic covalent diselenium bonds and noncovalent hydrogen bonds (H‐bonds) of the carboxyl group, makes SA a promising candidate for the development of supramolecular polymer networks through hierarchical self‐assembly. Selenium has been playing a pivotal role in optical materials for their unique properties.^[^ ^39^, ^40^ ^]^ Herein, we investigated SA as a selenium source to synthesize sulfur‐selenium rich polymers by copolymerizing with elemental sulfur and DVB, referred to as Poly(S‐SA‐DVB) (Figure 1c), a simple and easy way to vary the proportion of selenium in the resultant polymers. Due to its high sulfur selenium content, dynamic covalent S─S/S─Se bonds, and potential for functionalization, this type of material is expected to have excellent performance in the fields of optical lenses, viscosity, and heavy metal adsorption. The protocol enables successful IV reactions to take place at 100 °C with only a trace amount of H_2_S production. The resultant sulfur‐selenium rich polymers effectively shield UV irradiation with enhanced RI, adhesive properties, and excellent stability (Figure 1d). To the best of our knowledge, this is the first example to introduce SA as a selenium source for simultaneously integrating sulfur and selenium into polymeric materials through IV, innovating in both the reaction process and the resultant material itself.

## 2 Experimental Section

### 2.1 Synthesis of Poly(S‐SA 1:1)

Equal amounts of S_8_ (200 mg) and SA (200 mg) were added into a 25 mL reaction tube, then the reaction mixture was stirred at 100 °C for 12 h to obtain a dark yellow solid. Powder X‐ray diffraction (PXRD) and differential scanning calorimetry (DSC) results indicated that there is no remaining unreacted crystalline sulfur and SA in Poly(S‐SA).

### 2.2 Synthesis of Poly(SA‐DVB 1:1)

Equal amounts of S_8_ (200 mg) and DVB (200 mg) were added into a 25 mL reaction tube, then the reaction mixture was stirred at 100 °C for 12 h to obtain a dark yellow solid. PXRD and DSC results indicated that there is no remaining unreacted crystalline sulfur in Poly(SA‐DVB).

### 2.3 Synthesis of Poly(S‐SA‐DVB 1:1:1)

Equal amounts of S_8_ (200 mg), SA (200 mg), and DVB (200 mg) were added into a 25 mL reaction tube, then the reaction mixture was stirred at 100 °C for 12 h to yield a dark yellow solid. PXRD and DSC results indicated there is no remaining unreacted crystalline sulfur in Poly(S‐SA‐DVB).

### 2.4 Characterizations

**Figure 2:**
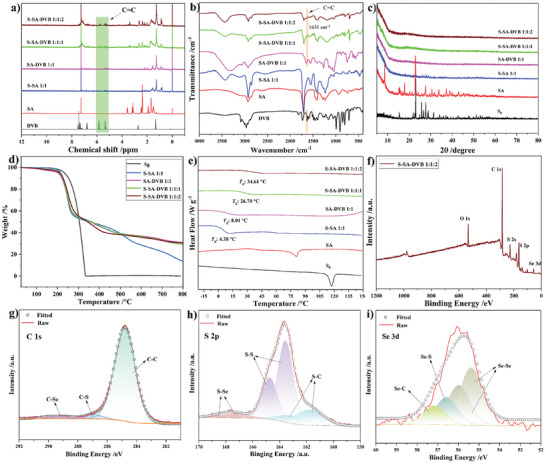
Shows the analysis of DVB, SA, Poly(S‐SA), Poly(SA‐DVB) and Poly(S‐SA‐DVB): a) ^1^H NMR; b) FT‐IR; c) PXRD; d) TGA; e) DSC; f) High‐resolution XPS spectra of Poly(S‐SA‐DVB 1:1:2); g) High‐resolution C 1s XPS spectra of Poly(S‐SA‐DVB 1:1:2); h) High‐resolution S 2p XPS spectra of Poly(S‐SA‐DVB 1:1:2); i) High‐resolution Se 3d XPS spectra of Poly(S‐SA‐DVB 1:1:2).

The obtained sulfur‐selenium rich polymers were comprehensively characterized, including nuclear magnetic resonance (NMR), infrared spectroscopy (IR), PXRD, thermogravimetric analysis (TGA), DSC, elemental analyzer (EA), gel permeation chromatography (GPC), and scanning electron microscope (SEM) (**Figure** **2**a; Figure S1, Supporting Information), e.g., Poly(S‐SA‐DVB 1:1:1) and Poly(S‐SA‐DVB 1:1:2), the ^1^H NMR spectra revealed that the C═C double bonds in DVB were almost entirely consumed. New peaks that emerged between 0.5 and 2.0 ppm could be attributed to the methylene peak subsequent to polymerization, recognized as one of the distinctive features of IV polymerization. Furthermore, a weaker broad peak was observed between 3.5 and 4.8 ppm, corresponding to the peak of ─SCH, supporting successful polymerization and incorporation of sulfur. The structural information and the bond conversion of Poly(S‐SA‐DVB) were further elucidated by Fourier transform infrared spectroscopy (FT‐IR, Figure 2b). The appearance of new peaks at 750 and 470 cm^−1^ evidenced the formation of C─S and S─S bonds.^[^ ^41^, ^42^ ^]^ PXRD analysis confirmed the phase of Poly(S‐SA‐DVB) as an amorphous powder (Figure 2c). There were no noticeable peaks of S_8_ and SA in the diffraction patterns of Poly(SA‐DVB) and Poly(S‐SA‐DVB) compared to their pristine ones. The thermal stabilities of Poly(S‐SA‐DVB) were investigated by using TGA (Figure 2d). It illustrates that the initial thermal decomposition temperature of this material was lower than S_8_, presumably due to the abundance of Se─Se and S─Se bonds, possessing lower bond energy than S─S bonds. Nevertheless, the initial decomposition temperature of the resultant materials were still maintained above 150 °C. Furthermore, the resultant polymers demonstrated a clear glass transition temperature (*T*_g_), increasing with a higher ratio of DVB presented. This could be attributed to the greater concentration of cross‐linking sites and a more compact cross‐linking network structure resulting from the increased DVB component (Figure 2e). Although higher DVB content typically increases crosslinking density and *T*_g_, an excessively high DVB ratio (e.g., 1:1:3 and 1:1:5) may lead to structural heterogeneity, unexpected behaviors or phase separation, resulting in a reduction in the effective crosslinking density and a corresponding decrease in *T*_g_. It is also possible that *T*_g_ decreases, which may be due to other unexpected behaviors of the ─COOH group during the reaction process.

GPC analysis was conducted to evaluate the molecular weight and distribution of the soluble fraction of the polymer, providing insights into the characteristics of the partially crosslinked structure. GPC analysis of the soluble fraction showed a relatively low molecular weight for both poly(S‐SA‐DVB 1:1:1) and Poly(S‐SA‐DVB 1:1:2) (Mn = 795 and 995, respectively), it is worth noting that the values here only indicate the molecular weight of the dissolved polymer, and so are not representative of the bulk material. This could be attributed to the temperature reduction from 160 to 100 °C during the reaction; however, the latter temperature range is still considerable high. The increased reaction temperature led to an increase in the initiator concentration (i.e., sulfur free radical concentration), resulting in an accelerated polymerization rate and a decreased polymerization degree. This implied that, as the sulfur free radical concentration increased, the rate of chain scission became slower than the rate of new molecule generation within a specific time interval. However, the obtained material demonstrated a favorable polydispersity index (PDI) of 1.2/1.4 (Figures S2 and S3 and Table S1, Supporting Information). It observed that, apart from a significant difference between the measured values of oxygen and the theoretical values, all other elements closely aligned with their respective theoretical values. This discrepancy in the oxygen values was likely attributable to the moisture trapped in the sample during the testing procedure (Table S2, Supporting Information). The morphology of the samples was characterized by SEM (Figure S5, Supporting Information). It evidenced a high degree of smoothness of the surfaces of the desired polymers, with no evidence of the ordered crystal habits that would be associated with sulfur bloom.

Further evidence supporting the structural information of Poly(S‐SA‐DVB) was presented through X‐ray photoelectron spectroscopy (XPS). As displayed in Figure 2f, the XPS survey results for Poly(S‐SA‐DVB) identified five peaks corresponding to O 1s, C 1s, S 2s, S 2p, and Se 3d, respectively. XPS spectra of S 2p exhibited five characteristic peaks at 167.8164.7, 163.6, 162.8, and 161.6 eV, respectively. The peaks located at 285.8 and 289.2 eV in the high‐resolution XPS spectra of C 1s further confirms the formation of C─S and C─Se bonds (Figure 2g). Moreover, the binding energy of 167.8 eV was ascribed to S−Se linkage, the binding energy of the S 2p3/2peak (163.6 eV) and S 2p1/2peak (164.7 eV) could be ascribed to S−S linkage, while the other two peaks with the binding energies of 162.8 and 161.6 eV could be raised from C─S bond (Figure 2h). The measured peaks of C─Se (57.2 eV), S─Se (56.6 eV), and Se─Se (55.3 and 55.9 eV) in Poly(S‐SA‐DVB) provided solid evidence for the existence of newly formed chemical bonds among the C, S and Se atoms in the S−Se−DVB polymers (Figure 2i).^[^ ^43^, ^44^, ^45^, ^46^ ^]^ It is worthy of noting that SA could potentially undergo self‐polymerization, polymerization with S_8_ or DVB, but even if this is occurring in the three‐component mixture, the single *T*_g_ observed suggests there is no significant phase separation. More interestingly, the protocol works for a broad scope of allylic organic comonomers (Scheme S1, Supporting Information). Thus, a range of crosslinking agents encompassing industrial waste and natural products, such as DIB, dicyclopentadiene (DCPD), limonene (LME) and myrcene (MYE), worked smoothly toward the desired polymers. All the resultant materials were fully characterized with the evidence of successful IV polymerization (Figure S1, Supporting Information).

## 3 Results and Discussion

### 3.1 Stability

**Figure 3:**
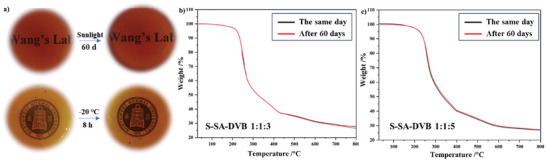
a) Stability comparison of Poly(S‐SA‐DVB 1:1:3) before and after 60 days of exposure to sunlight or Stability comparison of Poly(S‐SA‐DVB 1:1:5) before and after freezing at −20 °C for 8 h. b) The thermal stability of Poly(S‐SA‐DVB 1:1:3) was assessed pre‐ and post‐60 days. c) The thermal stability of Poly(S‐SA‐DVB 1:1:5) was assessed pre‐ and post‐60 days.

The desired polymers possessed good stability to endure extreme conditions and prolonged usage. To assess the stability of Poly(S‐SA‐DVB 1:1:3), Poly(S‐SA‐DVB 1:1:4), and Poly(S‐SA‐DVB 1:1:5), rigorous tests were conducted. No visible change in transparency and no signs of sulfur bloom occurred when the materials were subject to either freezing at −20 °C for 8 h or sunlight for 60 days at 30 °C (**Figure** **3**a; Figures S11–S16, Supporting Information). This thermal stability of the materials was also assessed by TGA of Poly(S‐SA‐DVB 1:1:3) and Poly(S‐SA‐DVB 1:1:5) immediately after the preparation and after 60 days of exposure, respectively, revealing identical thermal loss behavior between the two samples (Figure 3b,c). On this basis, we conducted DSC tests on Poly(S‐SA‐DVB 1:1:3) and Poly(S‐SA‐DVB 1:1:5) before and after freezing at −20 °C for 8 h and 80 °C for 16 h, The changes in the glass transition temperature of both are within the system error(±0.1–±1 °C), as shown in Figure S17 (Supporting Information). To our surprise, after being exposed to 10 W light at 365 nm for 6 h, the stability of the sample not only did not show any loss, but also improved, as shown in Figures S19 (Supporting Information). further demonstrating that the sulfur rich selenium polymer obtained from this system has excellent environmental stability.

### 3.2 Refractive Index Test

**Figure 4:**
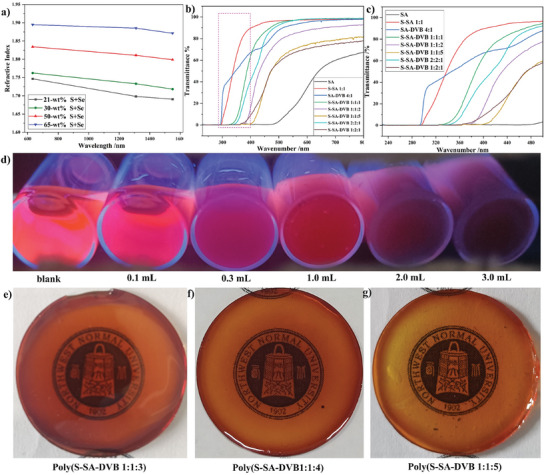
a) Refractive indices of poly(S‐SA‐DVB) terpolymers films of varying composition versus wavelength. b) UV–vis transmittance spectra of Poly(S‐SA‐DVB) solution with various material ratios. c) Photographs of fluorescent RB solution under 254 nm UV lamp, after adding different volumes of saturated poly(S‐SA‐DVB 1‐1‐2) DMF solutions to 3 mL of RB solution, respectively. d) UV–vis transmittance spectra of poly(S‐SA‐DVB) solutions with different material ratios at 200–500 nm. e) Photographs of a poly(S‐SA‐DVB 1:1:3) polymer film with a thickness of 1.02 mm showing good transparency under natural light. f) Photographs of a poly(S ‐SA‐DVB 1:1:3) polymer film with a thickness of 0.88 mm showing good transparency under natural light. g) Photographs of a poly(S‐SA‐DVB 1:1:3) polymer film with a thickness of 0.94 mm showing good transparency under natural light.

RI testing was consequently conducted on a series of Poly(S‐SA‐DVB) films with various ratios in order to assess the RI of these materials. The preparation details and the corresponding films are presented in the supplementary information (Figure S10, Supporting Information). The RI of the material was measured using a prism coupling instrument at three different wavelengths, namely 632, 1310, and 1550 nm (**Figure** **4**a; Table S4, Supporting Information). In the case of a mass ratio of 21% elemental sulfur and selenium, the RI of Poly(S‐SA‐DVB 1:1:5) at 632 nm approached 1.74, which is comparable to the RI (1.76) of the sulfur‐rich material generated from IV reaction containing 50% sulfur.^[^ ^26^ ^]^ With the sulfur and selenium content increasing to 30%, the RI of the resultant Poly(S‐SA‐DVB 1:1:3) increased to 1.76. Furthermore, a significant increasement of the RI of Poly(S‐SA‐DVB 2:1:2) up to 1.83 was achieved with a sulfur and selenium content of 50%. The RI of the desired sulfur‐selenium material was remarkably higher than that of Poly(S‐DIB 5:5) and similar to that of Poly(S‐DIB 7:3),^[^ ^23^ ^]^ demonstrating an effective enhancement of the desired material's RI by integrating SA into the IV polymerization. Moreover, an increase in the RI was also observed with the increase of SA content solely, evidenced by a high RI of 1.89 achieved with the sulfur and selenium contents reaching 65% in Poly(S‐SA‐DVB 5:3:2).

### 3.3 UV Shielding Performance Test

The UV shielding effect of materials obtained from SA and different ratios of S/SA/DVB was tested. As shown in Figure 4b,c, the SA monomer completely blocks ultraviolet light with limited transmission of visible light in the range of 400–500 nm. In comparison, Poly(S‐SA 1:1) and Poly(SA‐DVB 4:1) exhibited a significantly weaker UV shielding effect, only providing shielding below 300 nm. The introduction of DVB into the polymers enhanced the shielding effect. Thus, Poly(S‐SA‐DVB 1:1:1) was able to completely block the transmission of short and medium wavelength ultraviolet, while still allowing visible light (500–800 nm) to pass through with an efficiency of 95.05−98.61%. The complete shielding of long wavelength ultraviolet radiation is extremely harsh, but the remaining of shielding rate above 40% is a good achievement. Interestingly, the UV shielding performance of both Poly(S‐SA‐DVB 1:1:2) and Poly(S‐SA‐DVB 1:1:5) improves by increasing the DVB ratio in the polymerization reaction. Similarly, increasing the ratio of S_8_ and SA effectively improves the UV shielding performance of Poly(S‐SA‐DVB 2:2:1). These materials ensure 100% shielding against short and medium wavelength ultraviolet rays (200–320 nm), at the same time providing over 60% shielding for long wavelength ultraviolet rays (320–400 nm). However, increasing the SA ratio further improves the UV shielding effect of Poly(S‐SA‐DVB 1:2:1), the visible light transmittance is impeded. To further evaluate the UV shielding performance of this material, we prepared a saturated solution of Poly(S‐SA‐DVB 1:1:2) using DMF and a 100 ppm Rhodamine B solution. Different volumes of saturated solutions were added to the Rhodamine B solution and placed in the dark conditions. The samples were then irradiated with 254 nm ultraviolet light. The experiment compared the blank sample with different concentrations of Poly(S‐SA‐DVB 1:1:2). It was observed that the brightness of the solution decreased with the concentration of Poly(S‐SA‐DVB 1:1:2) increasing, indicating an effective shielding effect of the desired materials against ultraviolet light (Figure 4d).

### 3.4 Adhesivity

**Figure 5:**
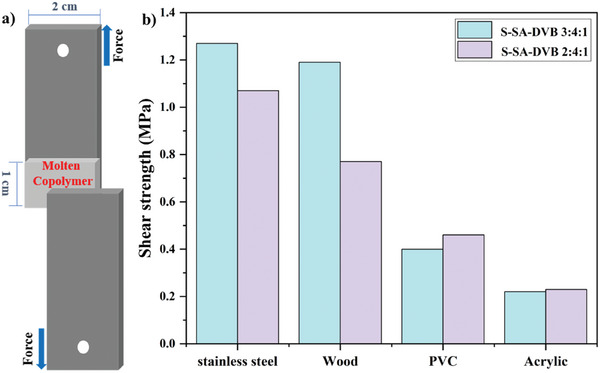
a) Schematic representation of the adhesion procedure. b) Comparison of shear strength of Poly(S‐SA‐DVB) on five different types of surfaces.

The sulfur‐selenium polymers displayed adjustable viscosity by changing the selenium ratio within the polymer. Therefore, by introducing the proportion of SA in polymer materials ≈50% or over with reduced proportion of DVB, the resultant polymers could easily be transitioned into a molten state by heating, displaying thermoplastic properties and resulting in viscosity. This is presumably due to the H‐bonds formed among those abundant carboxyl groups with polyhydroxy groups on the surface of the polymers (e.g., glass slice, PVC, and acrylic).^[^ ^12^ ^]^ Thus, the Poly(S‐SA‐DVB 3:4:1) and Poly(S‐SA‐DVB 2:4:1) were transformed into liquid copolymers at 140 °C, which were consequently added dropwise to the surface of a glass slice. The glass slice is then pressed together with another clean glass slide to form a glued area of 1 cm × 2 cm of 40 µm thickness (**Figure** **5**a). These two adhered glass slices were able to lift weights of 20 kg without any displacement in their glued area in the dynamic lifting experiment, thus revealing their ultrahigh adhesive strength (Video S1, Supporting Information). However, there have been instances of breakage occurring outside the adhesive surface during shear force testing (Figure S20, Supporting Information). Unfortunately, no exact shear force data for the glass substrate has been obtained. As a result, we conducted a series of tests using stainless steel, wood, acrylic board, and polyvinyl chloride (PVC) board as alternative substrates (Figure S21, Supporting Information). The adhesivity on stainless steel distinguished from the other substrates, the resultant copolymer exhibited a remarkable and excellent adhesive capability with a shear strength of 1.27 MPa (Figure 5b). It is also worth noting that COOH groups help form H‐bonds with materials with ─OH groups on their surfaces, thereby increasing the viscosity of materials such as glass, PVC, and acrylic. Poly (S‐SA‐DVB 2:4:1) has significantly better adhesion to PVC and Acrylic than Poly (S‐SA‐DVB 3:4:1). But the surfaces of stainless steel plates and wood have almost no ─OH, so there is no hydrogen bonding to assist in the enhancement effect. Although S‐SA‐DVB (2:4:1) contains a higher COOH content, its greater crosslinking density limits polymer chain mobility, reducing effective substrate interaction under shear forces. Conversely, S‐SA‐DVB (3:4:1) exhibits improved adhesion due to its more flexible polymer network, which facilitates better substrate wetting and contact. In comparison to commercial glue, poly(S‐SA‐DVB 3:4:1), and poly(S‐SA‐DVB 2:4:1) afforded enhanced adhesive forces on four different tested surfaces.^[^ ^12^ ^]^ Compared to Poly(S‐DIB) derivatives, our material has high adhesive forces.^[^ ^47^ ^]^ Compared with the sulfur rich polymer of 50% garlic essential oil, poly(S‐SA‐DVB 3:4:1), and poly(S‐SA‐DVB 2:4:1) exhibits high adhesive forces.^[^ ^48^ ^]^

### 3.5 Possible Mechanism

**Figure 6:**
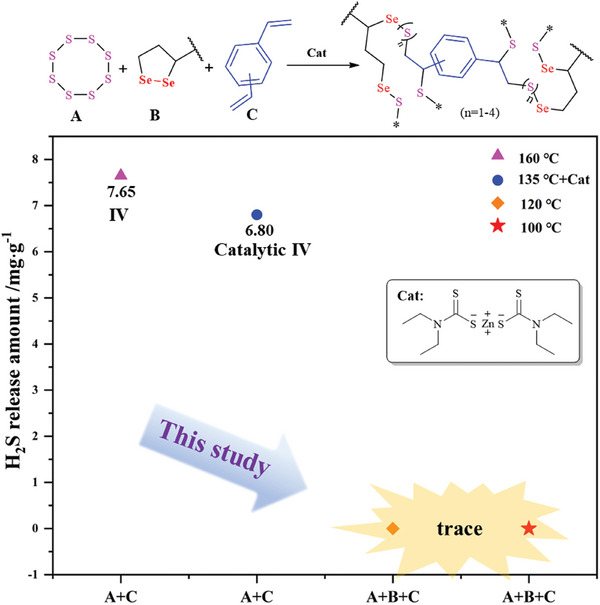
Comparison of hydrogen sulfide (H_2_S) generated during IV, catalytic IV and selenium copolymer IV.

Discussing given the similarity of thermal conditions for normal IV polymerization applied in this protocol, the production of toxic byproduct of H_2_S is a key measurement. About 7.65 mg g^−1^ of H_2_S was generated by traditional IV (DVB50‐S50 reacted at 160 °C with a total mass of 200 mg) and 6.80 mg g^−1^ of H_2_S was generated from catalytic IV (DVB50‐S50 reacted at 135 °C with a total mass of 200 mg in the presence of 3 wt.% catalyst) using lead acetate solution. However, only trace amounts of H_2_S were detected from the IV reaction incorporating the third component SA, while reducing the reaction temperature to 100 °C (as shown in **Figure** **6**; Table S3, Supporting Information). Additionally, it is important to note that the bond energy of Se─Se (172 kJ mol^−1^) is significantly lower than that of S─S bond (240 kJ mol^−1^). Based on these observations, we propose the following mechanism for this process. First, SA undergoes a Se─Se bond fracture leading to the formation of Se free radical. The sulfur and selenium radicals generated under the reaction conditions interact with the unsaturated double bonds of the monomers, leading to the formation of a polysulfide‐selenide copolymer (Scheme S2, Supporting Information).

## 4 Conclusion

In summary, a series of sulfur‐rich polyselenides were synthesized using a solvent‐free approach, enabling the tunability of application performance through the adjustment of material ratio. The current protocol is especially significant for its environmental sustainability, as it minimizes H_2_S production by facilitating reactions at lower temperatures compared to the traditional IV processes. The resultant material provides exceptional UV protection while maintaining over 80% visible light transmission, ensuring high transparency across the entire visible light spectrum. Furthermore, it exhibits superior optical properties, boasting a RI of up to 1.89. The material demonstrates remarkable thermal stability, maintaining its properties even under extreme conditions, such as exposure to −20 °C for 8 h or at room temperature for 60 days under sunlight and ambient air. Additionally, we have identified that the material possesses viscosity, allowing it to serve as both an optical window and an adhesive in compact instruments. As a result, this material demonstrates substantial potential as a reliable and flexible optical medium, offering a cost‐effective alternative to expensive inorganic optical components currently used in high‐end instrument applications. The abundant S─S/S─Se bonds in this material indicate that its properties can be leveraged through dynamic covalent interactions, enabling its degradation and modification into smaller molecules. This process aims to facilitate cyclic closure between the monomer and the material, partially addressing the challenge of efficiently degrading the polymer.

## Conflict of Interest

The authors declare no conflict of interest.

## Author Contributions

J.J. performed conceptualization, formal analysis, investigation, methodology, visualization, and wrote – original draft. Y.C. performed investigation, methodology, validation, and wrote – original draft. Y.G. performed methodology, validation, and formal analysis. X.X. performed formal analysis and validation. T.Q. performed formal analysis, software, and validation. X.W. performed validation and software. X.‐C.W. performed project administration. X.W. performed conceptualization, supervision, wrote – original draft, and wrote – review and editing. T.H. performed conceptualization, supervision, wrote – original draft, and wrote – review and editing. Z.‐J.Q. performed conceptualization, supervision, acquired funding acquisition, resources, and wrote – review and editing.

## Supporting information



Supporting Information

Supporting Information VideoS1

## Data Availability

The data that support the findings of this study are available in the supplementary material of this article.
