# Supplementary material for: Dynamic Covalent Sulfur‐Selenium Rich Polymers via Inverse Vulcanization for High Refractive Index, High Transmittance, and UV Shielding Materials
Source: Macromol Rapid Commun. 2025 Jan 15;46(6):2400998. doi: 10.1002/marc.202400998 (PMC11925326; doi:10.1002/marc.202400998)
Supplement: Supplementary file 1 — Supporting Information [file MARC-46-2400998-s002.docx]

**Supporting Information**

**Dynamic Covalent Sulfur-Selenium Rich Polymers Via Inverse Vulcanization for High Refractive Index, High Transmittance, and Excellent UV Shielding Materials**

Jinhong Jia,^1^ Yao Chai,^1^ Xingwei Xun,^1^ Yan Gao,^2^ Tongsen Qiao,^3^ Xiong Wang,^3^ Xi-Cun Wang,^1*^ Tom Hasell,^4*^ Xiaofeng Wu,^1,4*^ and Zheng-Jun Quan,^1*^

^1^ College of Chemistry and Chemical Engineering, Gansu International Scientific and Technological Cooperation Base of Water-Retention Chemical Functional Material, Northwest Normal University, Lanzhou, Gansu, 730070, P.R.China

^2^ Lanzhou Petrochemical Branch of China National Petroleum Corporation, Lanzhou, Gansu, 730060,P.R.China

^3^ Lanzhou Petrochemical Research Center, PetroChina Petrochemical Research Institute, Lanzhou, Gansu, 730060,P.R.China

^4^ Leverhulme Research Centre for Functional Materials Design, Materials Innovation Factory, and Department of Chemistry, University of Liverpool,Liverpool,L697ZD,UK

**Table of Contents**

1. **Chemicals and Materials S3**
2. **Instrumentation used for characterization S4**
3. **Preparation of raw material S5**
4. **Preparation of materials S6**
5. **Substrate expansion S7**
6. **GPC spectra and results S8**
7. **EA analysis results S9**
8. **SEM analysis results S10**
9. **Solubility test S11**
10. **Possible mechanisms S12**
11. **Detection of H_2_S S13**
12. **Transparency research S14**
13. **Preparation of refractive index testing materials S15**
14. **Stability test S16-S23**
15. **Adhesion performance S24**

**Experimental Procedures**

**Chemicals and Materials：**

Sulfur (S_8_, sublimed powder, ≥99.5%, Aldrich), 1,3-Divinylbenzene (DVB, >55.0%, Aldrich), Myrcene (MYE, >90.0%, Aladdin), Limonene (LME, 95.0%, J&K Scientific Ltd.), Ethyl 6,8-dichlorooctanoate (>90.0%, Leyan), Selenium (≥96.0%, J&K Scientific Ltd),Sodium hydroxide (NaOH, ≥96.0%, Aladdin),Ethyl 6,8-dichlorooctanoate(96%, Energy Chemical),Charcoal active granular (Fuchen (Tianjin) Chemical Reagent Co., Ltd), Hexadecyl trimethyl ammonium bromide(≥93.0%,Shanghai Chemical Reagent Procurement and Supply Station), Sodium borohydride(NaBH_4_, ≥96.0%,Shanghai Shanpu Chemical Co., Ltd), Ethanol (95.0%,Sigma-Aldrich),Chloroform-d (CDCl_3_, 99.8 ato m%D, stab.mit Ag, cont.0.03V/V% TMS, Ningbo Cuiying Chemical Technology Co., Ltd), Tetrahydrofuran (THF, >99.9%, Aladdin),Dichloromethane(DCM, ≥99.5%,Energy Chemical),N,N-Dimethylformamide (DMF,≥99.5%, Tansoole), Toluene(TL, ≥99.5%, Chengdu Kelong Chemical Co., Ltd),Trichloromethane(CHCl_3_, ≥99.0%, Chengdu Kelong Chemical Co., Ltd),Acetone(AC, ≥99.5%, Chengdu Kelong Chemical Co., Ltd),Dimethyl sulfoxide(DMSO, ≥99.5%,Ke An Long Bo Hua (Tianjin) Pharmaceutical Chemistry Co., Ltd)，1，4-Dioxane(99.5%,Tianjin Fuyu Fine Chemical Co.,Ltd),Rhodamine B(technical grade, Energy Chemical),Zinc diethyldithiocarbamate (99%+, Tansoole),

**Instrumentation used for characterization：**

Nuclear Magnetic Resonance (NMR, Varian Mercury Plus 400 and Agilent DD2-600 MHz, USA) provides detailed information about molecular structure, and chemical reactions processes. In general, CDCl_3_ was used as the solvent as clarified otherwise.

Fourier-Transform Infrared (FT-IR, Digilab FTS-3000, USA) spectra exhibit the changes of chemical bonds before and after polymerization.

Powder X-ray Diffraction (PXRD, Rigaku D/Max-2200PC, Japan) patterns of S_8_ and polymers were characterized using Cu Kα radiation (λ =1.5418 Å) at 40 kV, 100 mA. Scanning range was from 5° to 80° .
Thermogravimetric Analysis (TGA, Mettler Toledo TGA/DSC1, Switzerland) was performed in a N_2_ flow from 25 °C to 800 °C at a rate of 10 °C min^-1^ .

Differential Scanning Calorimetry (DSC, TAQ2000, USA) was conducted at constant heating and cooling rates of 5 °C min^-1^ under N_2_ atmosphere in the range from -50 °C to 150 °C.

Elemental Analyzer (EA) was measured on an Elementar vario el cube.

UV-Vis absorption spectra were recorded on an Agilent 8453 UV-Vis spectrophotometer (JAPAN)

X-ray photoelectron spectroscopy (XPS) was performed in a Thermal ESCALAB Xi + Scanning electron microscope (SEM): In suit coating samples’ surfaces were observed on Hitachi SEM S4800. Energy dispersive spectroscopy (EDS): Elemental distribution maps of polymer-coated filter paper were recorded on Hitachi SEM S4800.

Gel permeation chromatography (GPC, Waters 1515-2414-2707, USA) uses polystyrene (PS) as the standard, THF as the solvent, and uses PL gel mixed C chromatographic column to analyze the polymer's potential at 35 °C for the soluble fraction.

Universal mechanical testing machine (CMT6103 Manufacturer MTS Meters Industrial System)

Prism coupling instrument (Metricon, 2010/M)

**Preparation of raw material^[1]^:**

1. In the synthesis of Na_2_Se_2_ alkaline aqueous solution and organic diselenide, 2 g (0.05 mol) of NaOH solid is dissolved in 25 mL of water. This is followed by the addition of 3.95 g (50 mmol) of selenium powder and 100 mg of hexadecyl trimethyl ammonium bromide. To dissolve 0.25 g (6.6 mmol) NaBH_4_ and 0.2g NaOH solids, 5 mL of water is added and the solution is cooled using an ice bath. This solution is then added to the selenium solution while stirring and under the protection of N_2_, and allowed to react at room temperature. The reaction is then completed by heating at 90 ℃ for half an hour, resulting in the characteristic brownish red Na_2_Se_2_ alkaline aqueous solution. This solution can be used for the next step of the synthesis of two Selenide without any treatment.

2. Put 6.01 g (25 mmol) of ethyl 6,8-dichlorooctanoate into a 250 mL three-necked flask. Next, 10 mL of 95% ethanol, 2 g (50 mmol) of sodium hydroxide, and 38 mL of water were sequentially added. Heat to 50 °C and react for 2 hours. A sodium selenide aqueous solution was added dropwise, the temperature was controlled at 70 °C., and the dropwise addition time was about 2 hours. After the dropwise addition, the reaction was continued at this temperature for 3 hours. Lower the temperature to 40 °C, add 4 g of activated carbon, stir for 30 minutes, and filter while hot to remove the activated carbon. Add water to the filtrate to make up to 350 mL, and cool to 0 °C. Add 5% mass concentration of dilute hydrochloric acid solution under rapid stirring, acidify dropwise, adjust the pH to 2, and precipitate a large number of brown solids. Filter and dry to obtain the final product Selenium octanoic acid (SA).

**Preparation of materials**

**Scheme S1** Preparation of Poly (SA), Poly (S-SA), Poly (SA-XX) and Poly (S-SA-XX)

**Results and Discussion**

**Substrate expansion：**

**
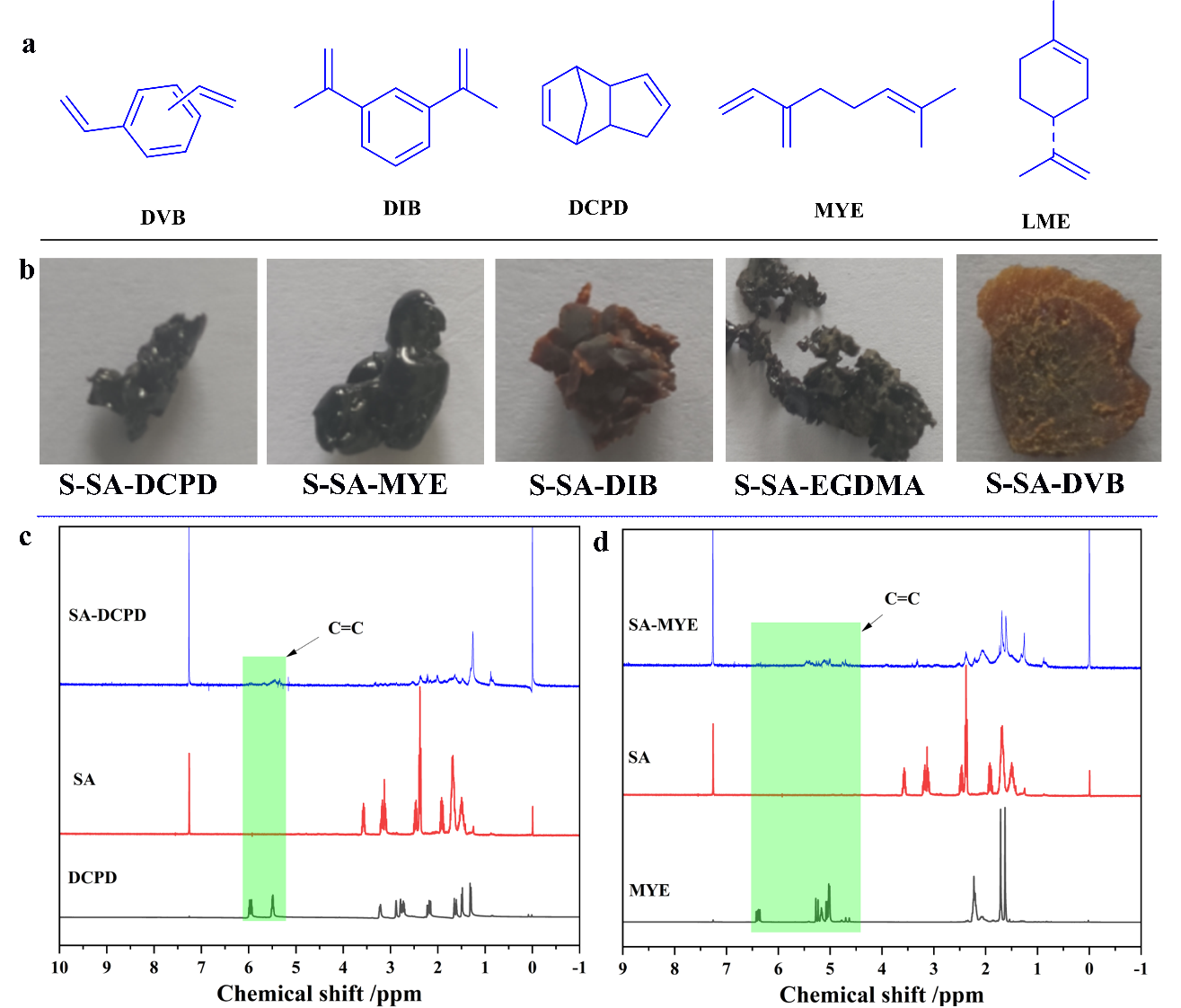
**

**Figure.S1** a) Expanding substrate; b) Specific phenomenon diagram of Poly (S-SA-XX); c) Poly (S-SA-DCPD) nuclear magnetic data analysis; d) Poly (S-SA-MYE) nuclear magnetic data analysis

**GPC spectra and results:**

Grind the obtained polymer into powder, then dissolve 20 mg of Poly (S-SA-DVB 1:1:1) and Poly (S-SA-DVB 1:1:2) in 4 mL of THF, stir and filter for later use. Filter with a 0.22 μ m filter head before testing to obtain the test solution. The actual test concentrations of Poly (S-SA-DVB 1:1:1) and Poly (S-SA-DVB 1:1:2) were 2.53 and 2.43 mg/mL, respectively


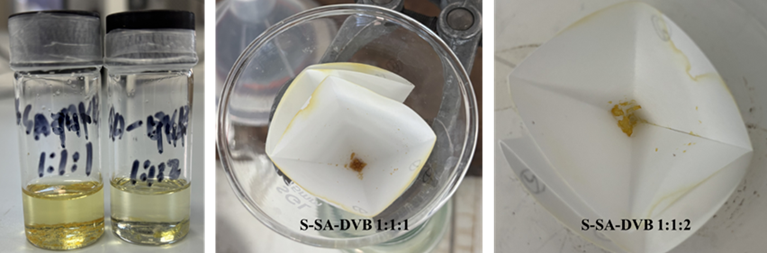


**Figure.S2** Characteristics of Poly (S-SA-DVB 1:1:1) and Poly (S-SA-DVB 1:1:2) before and after filtration


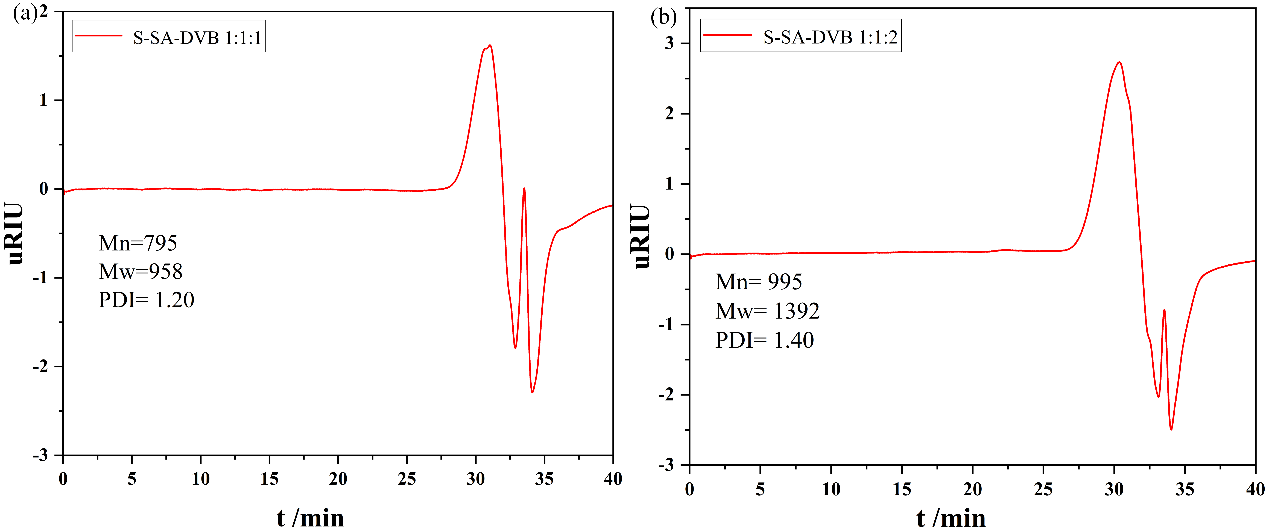


**Figure.S3** Analysis of GPC spectra of Poly(S-SA-DVB 1:1:1) and Poly(S-SA-DVB 1:1:2)

Relative to the standard polystyrene, the following molar mass averages (g mol^-1^ ) and PDI were calculated:

**Table S1**. GPC results of Poly(S-SA-DVB 1:1:1) and Poly(S-SA-DVB 1:1:2).

| Sample | *Mn*(g/mol) | *Mw*(g/mol) | PDI |
| --- | --- | --- | --- |
| Poly(S-SA-DVB 1:1:1) | 795 | 958 | 1.20 |
| Poly(S-SA-DVB 1:1:2) | 995 | 1392 | 1.40 |


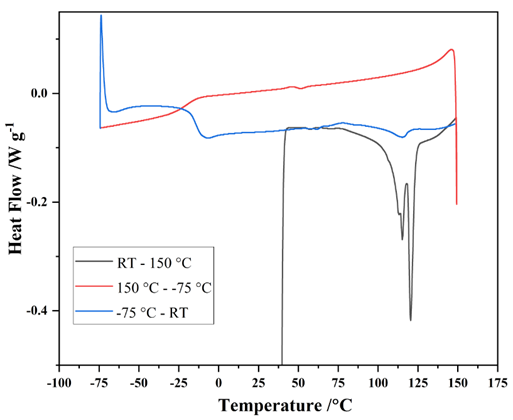


**Figure.S4** DSC curve of Poly (S-SA-DVB 1:1:1)

**EA analysis results:**

**Table S2**. EA results of Poly(S-SA 1:1).

|  | **N(%)** | **C(%)** | **H(%)** | **S(%)** | **O(%)** |
| --- | --- | --- | --- | --- | --- |
| **Poly(S-SA 1:1)** | 0.09 | 17.94 | 2.463 | 47.479 | 10.700 |
| **Theoretical value** | 0 | 16 | 2.33 | 50.00 | 5.33 |

Note: The reason for using Poly (S-SA 1:1) as a template for EA testing is because the DVB content is only 55%

**SEM analysis results:**


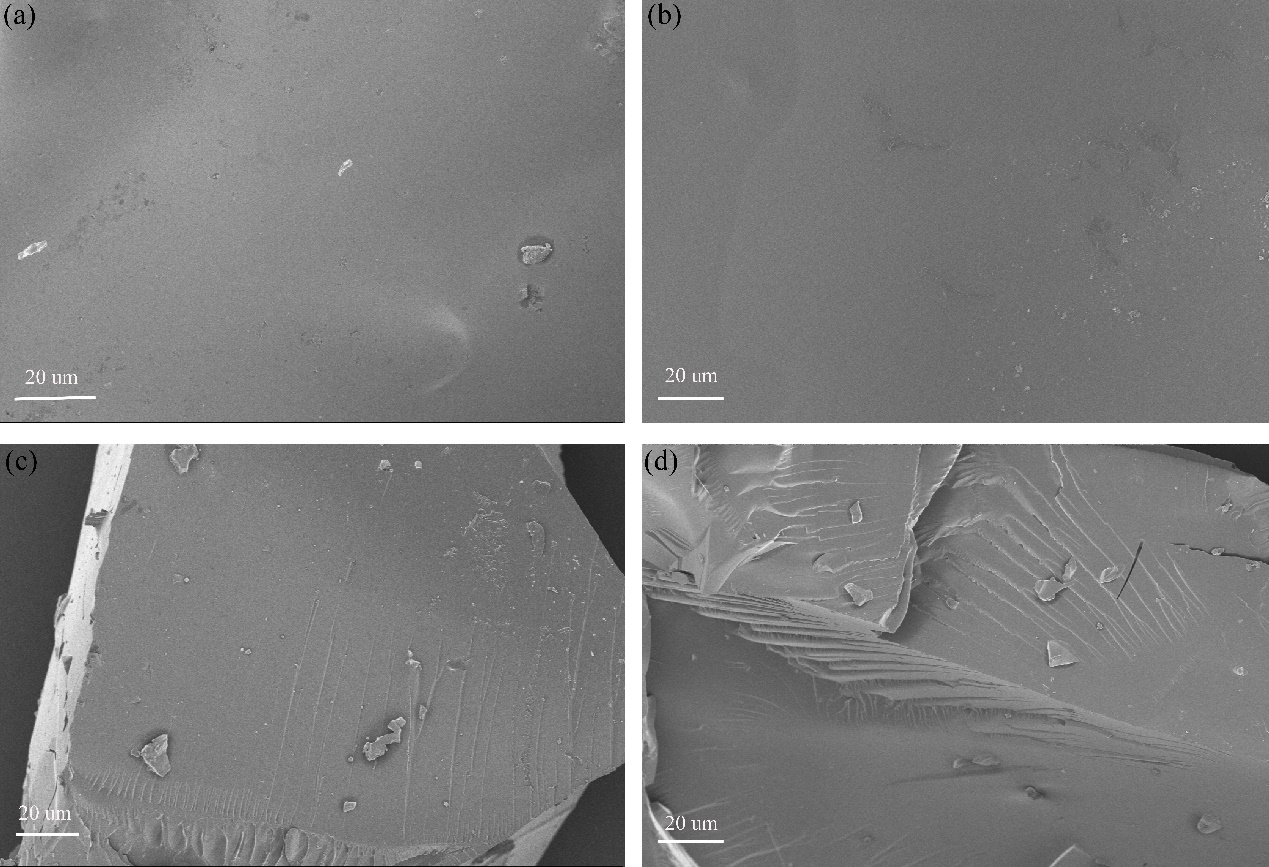


**Figure.S5**. SEM spectrum of Poly(S-SA-DVB). a) :Poly( S-SA 1:1); b)Poly( SA-DVB 1:1); c): Poly(S-SA-DVB 1:1:1); d) Poly(S-SA-DVB 1:1:2)

**Solubility test：**


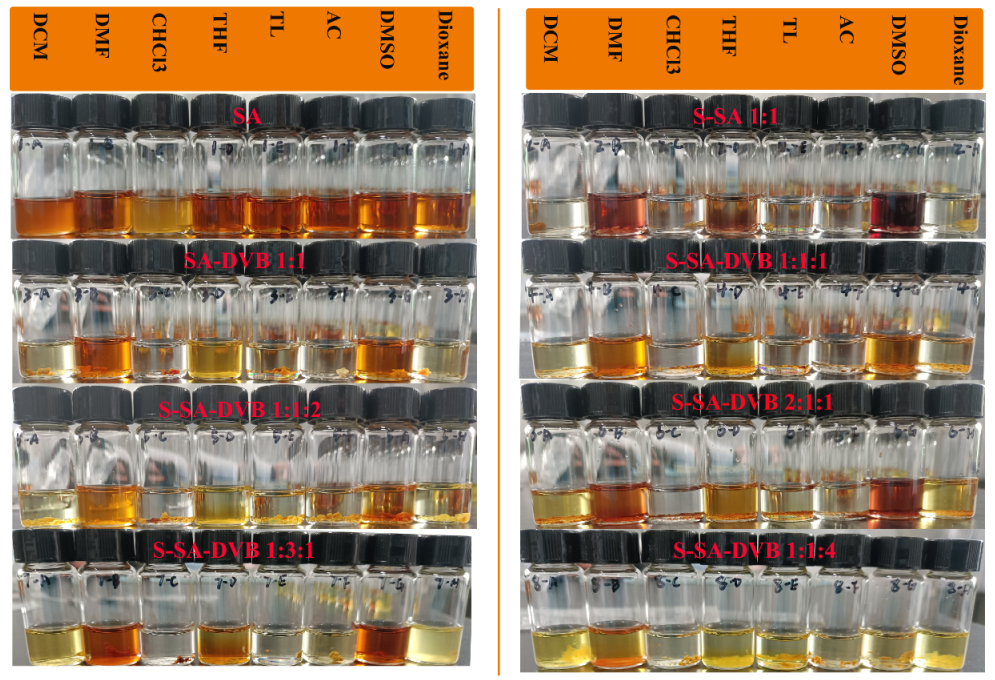


**Figure.S6** Solubility Test of Poly(S-SA-DVB) with Different Proportions in Different Solvents


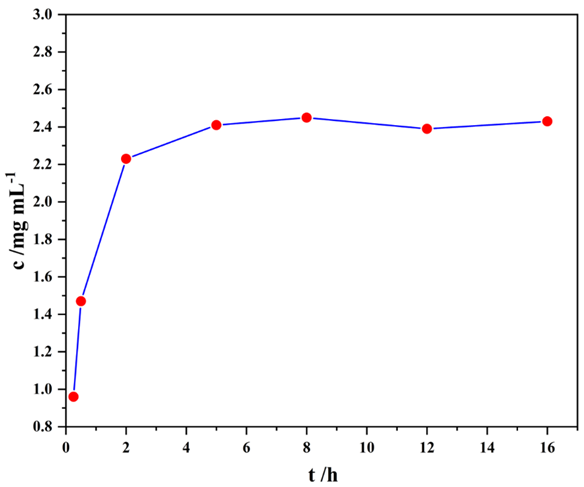


**Figure.S7** The solubility curve of poly (S-SA-DVB 1:1:2) over time

**Possible mechanisms：**

**Scheme S2** Possible mechanisms

**H_2_S detection：**

**Table S3**.H_2_S results of Poly(S-SA-DVB).

Principle：Pb(Ac)_2_+H_2_S=PbS+2HAc PbS is a black precipitation

| **Sample** | **T /℃** | **Cat** | **H_2_S /mg g-1** |
| --- | --- | --- | --- |
| A+B+C | 100 | / | trace |
|  | 120 | / | trace |
| A+C | 135 | 3% | 6.80 |
|  | 160 | / | 7.65 |

**Note: 100 mg each for A, B, and C**

**Transparency research:**


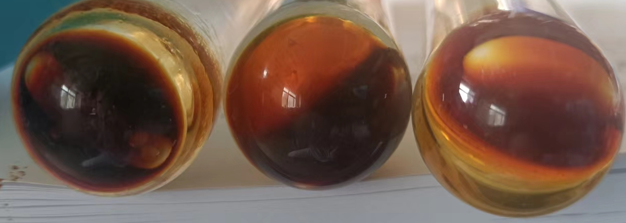


Poly(S-SA-DVB 5-1-1) Poly(S-SA-DVB 5-1-2) Poly(S-SA-DVB 5-1-3)

**Figure.S8** Comparison of Transparency of Polymer Materials with Different DVB Content


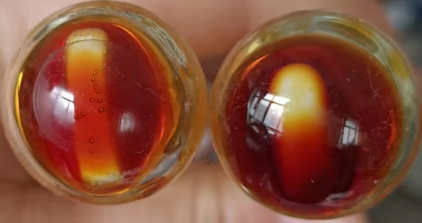


**Figure.S9** Comparison of Transparency of Polymer Materials with Different SA Content

**Preparation of refractive index testing materials:**


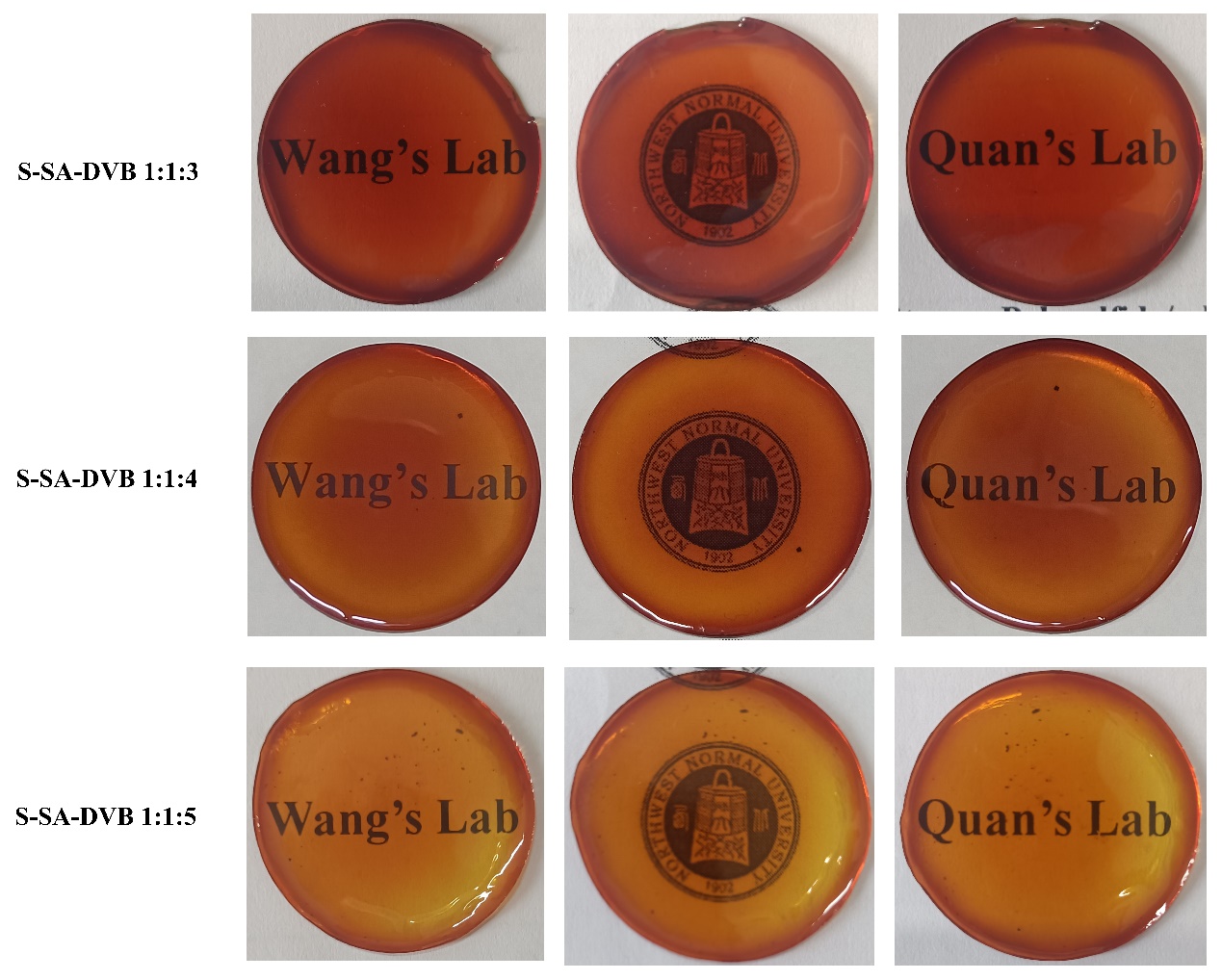


**Figure.S10** Add S_8_, SA, and DVB with different material ratios to a 5 mL transparent screw-shaped glass bottle in a single container. Heat the mixture at 120 °C until it forms a transparent state, which takes approximately 0.5 hours. Subsequently, pour the heated mixture into a high-temperature resistant silicone mold and allow it to cure at 120 °C for 3 hours.

**Table 4**. The specific refractive indices of polymers with varying sulfur and selenium contents were measured at wavelengths of 632 nm, 1310 nm and 1550 nm.

|  | **632 nm** | **1310 nm** | **1550 nm** |
| --- | --- | --- | --- |
| **21wt% S+Se** | 1.7465 | 1.6982 | 1.6907 |
| **30wt% S+Se** | 1.7624 | 1.7329 | 1.7183 |
| **50wt% S+Se** | 1.8342 | 1.8109 | 1.7988 |
| **65wt% S+Se** | 1.8947 | 1.8852 | 1.8712 |

**Stability test:**


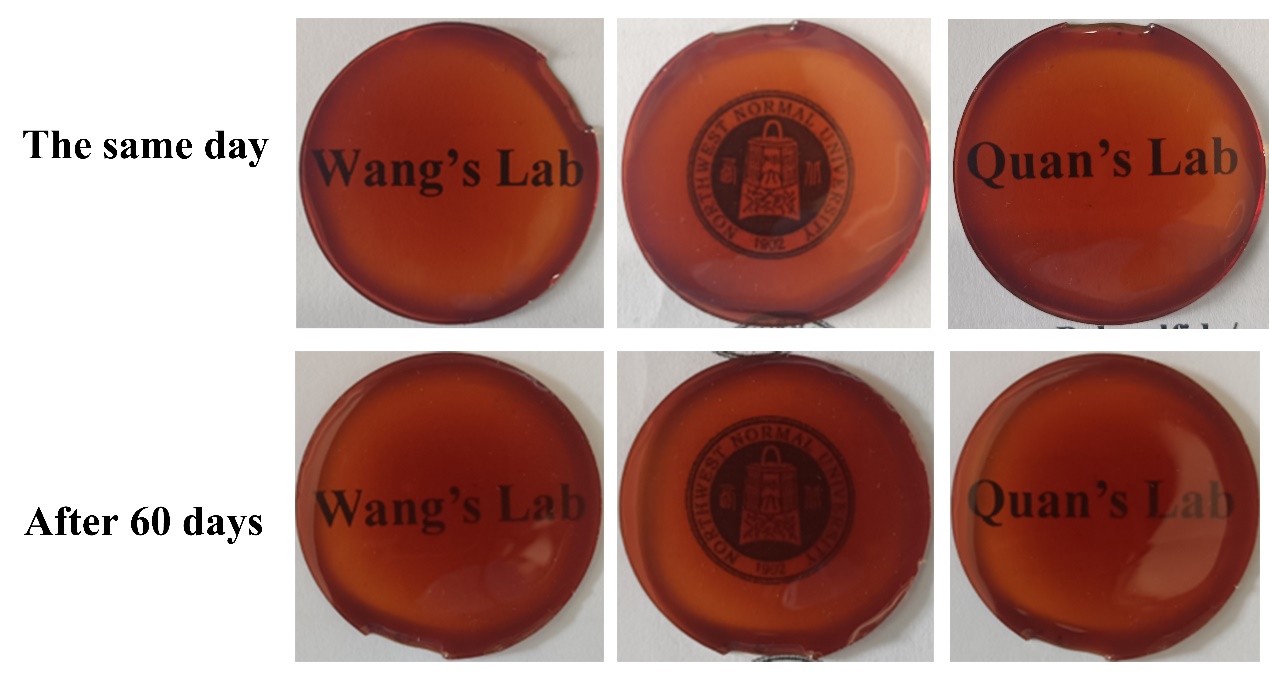


**Figure.S11**. Comparison of optical transparency between Poly(S-SA-DVB 1-1-3) on the day of preparation and 60 days later


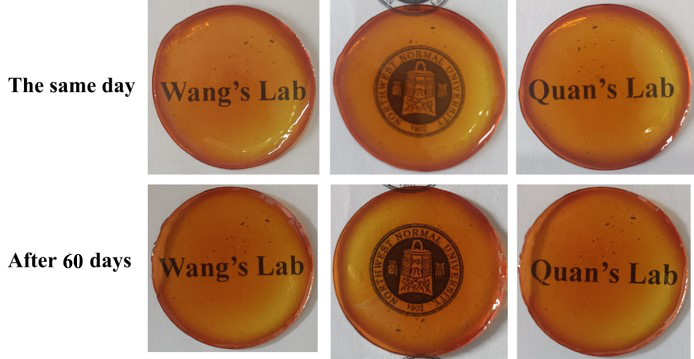


**Figure.S12**. Comparison of optical transparency between Poly(S-SA-DVB 1-1-4) on the day of preparation and 60 days later


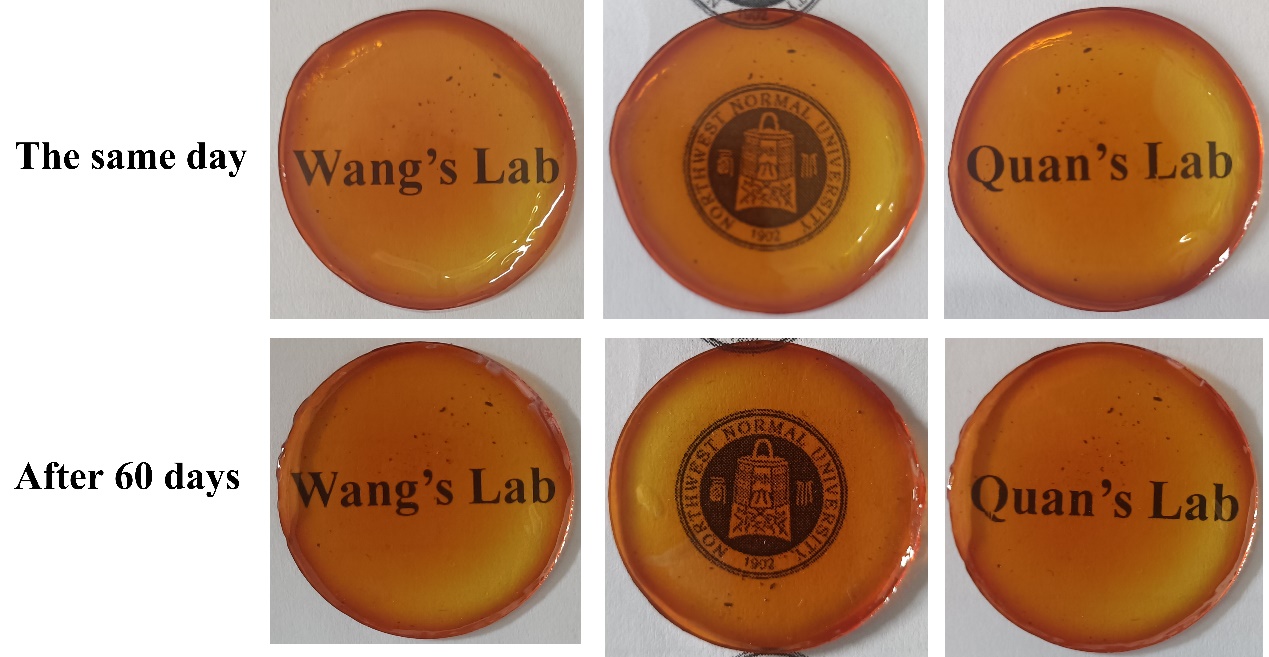


**Figure.S13**. Comparison of optical transparency between Poly(S-SA-DVB 1-1-5) on the day of preparation and 60 days later


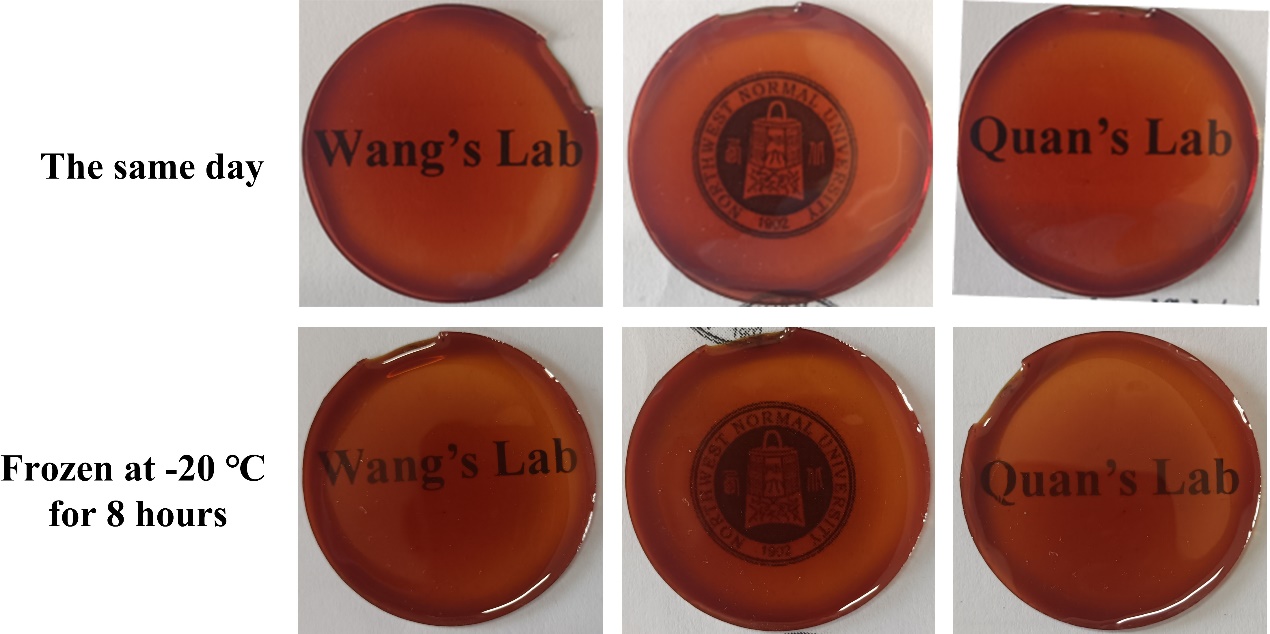


**Figure.S14**. Comparison of optical transparency between Poly(S-SA-DVB 1-1-3) at room temperature and -20 ℃ after 8 hours of freezing


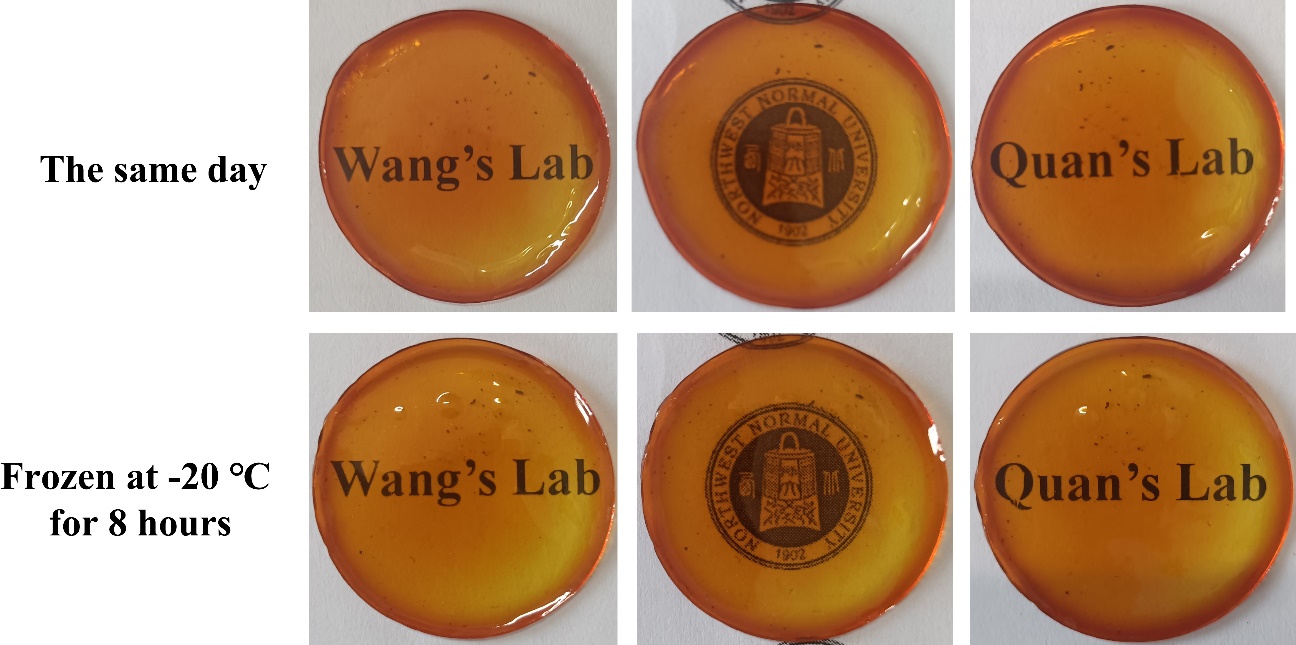


**Figure.S15.** Comparison of optical transparency between Poly(S-SA-DVB 1-1-4) at room temperature and -20 ℃ after 8 hours of freezing


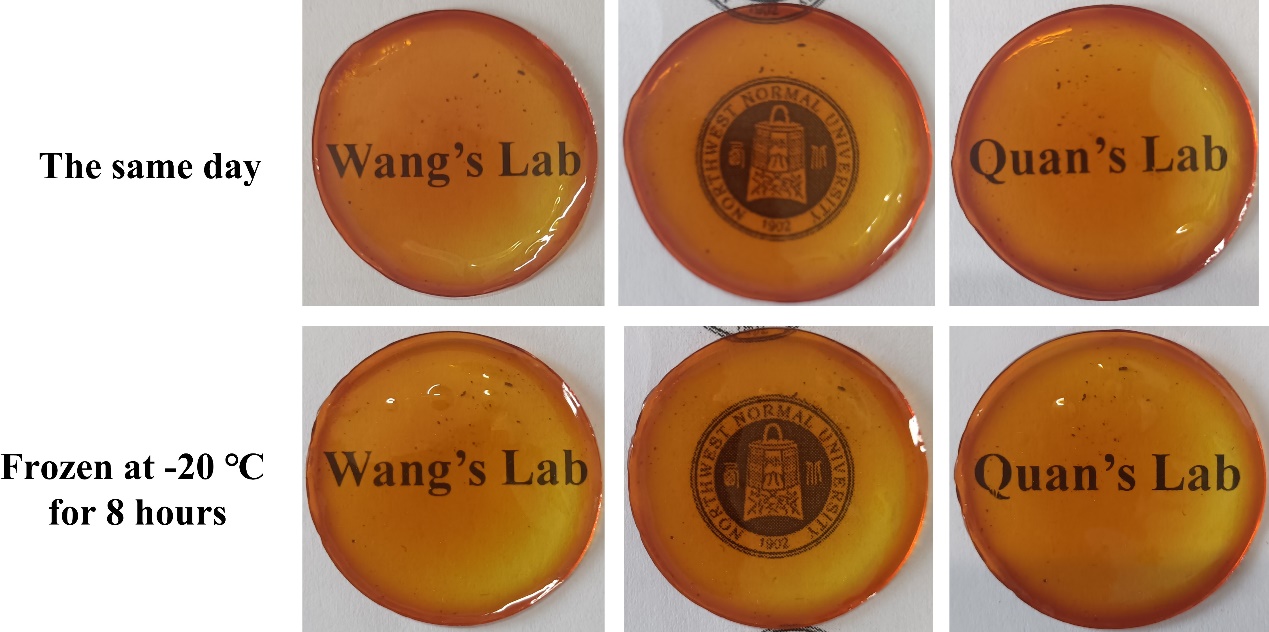


**Figure.S16**. Comparison of optical transparency between Poly(S-SA-DVB 1-1-5) at room temperature and -20 ℃ after 8 hours of freezing

**Stability**


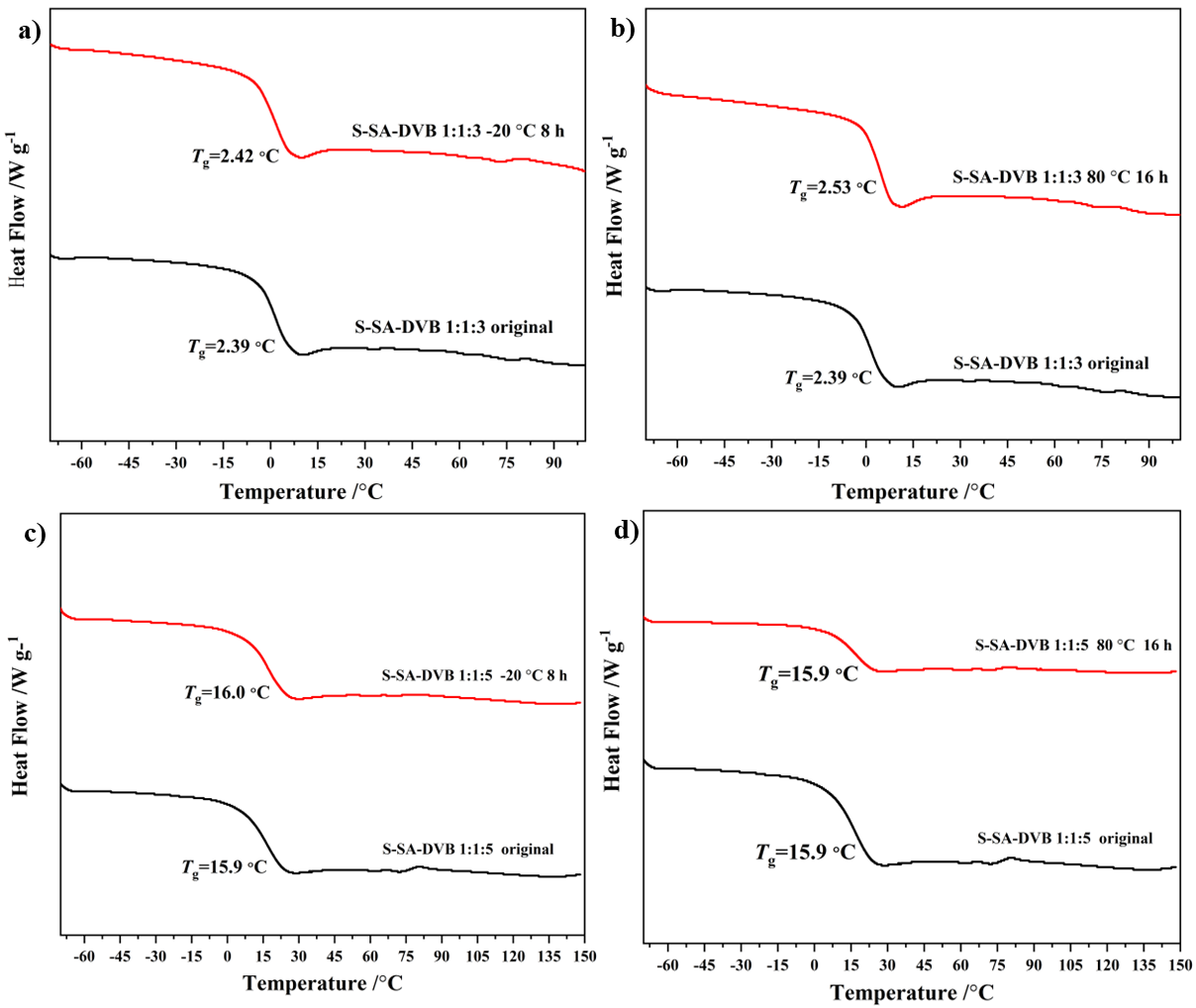


**Figure.S17** a) *T*_g_ changes of Poly(S-SA-DVB 1:1:3) before and after freezing at -20 ℃ for 8 hours.b) *T*_g_ changes of Poly(S-SA-DVB 1:1:3) before and after heating at 80 ℃ for 16 hours. c) *T*_g_ changes of Poly(S-SA-DVB 1:1:5) before and after freezing at -20 ℃ for 8 hours.d) *T*_g_ changes of Poly(S-SA-DVB 1:1:5) before and after heating at 80 ℃ for 16 hours.

On this basis, we conducted DSC tests on Poly(S-SA-DVB 1:1:3) and Poly(S-SA-DVB 1:1:5) before and after freezing at -20 ℃ for 8 hours to observe whether there was a change in *T*_g_, as shown in Figures 4d and 4f. It can be seen that after freezing, the *T*_g_ of Poly(S-SA-DVB 1:1:3) only increased by 0.03 ℃ (2.39 ℃ → 2.42 ℃); After freezing, the *T*_g_ of Poly(S-SA-DVB 1:1:5) increased by 0.1 ℃ (15.9 ℃ → 16.0 ℃); The changes in the glass transition temperature of both are within the system error(±0.1 °C - ±1 °C), which may be caused by the error of the testing instrument itself, indicating that the material's performance will not be affected even under extreme conditions of -20 ℃. Previously, we demonstrated that the transparency and stability of the material remained unchanged after 60 days of exposure to sunlight, indicating its good stability. However, considering the temperature conditions in different regions and the instrument's own heat generation, we heated Poly(S-SA-DVB 1:1:3) and Poly(S-SA-DVB 1:1:5) to 80 ° C and held them for 16 hours. Through DSC testing of the material before and after heating, we found that the *T*_g_ of Poly(S-SA-DVB 1:1:3) only changed by 0.14 ° C (2.39 ° C → 2.53 ° C), while the *T*_g_ of Poly(S-SA-DVB 1:1:5) remained unchanged (Figures 4e and 4g), further demonstrating that the sulfur rich selenium polymer obtained from this system has excellent environmental stability.


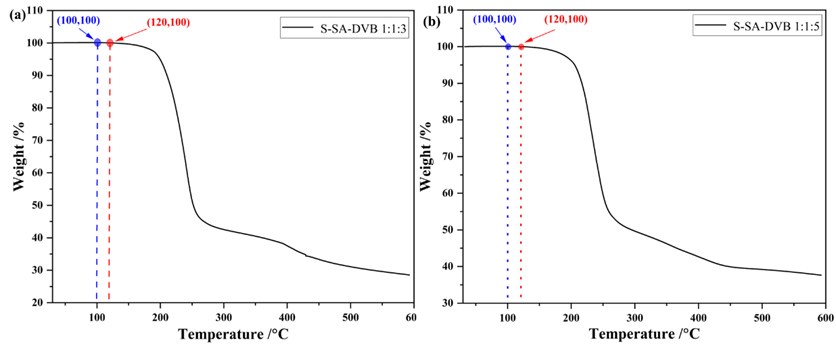


**Figure.S18** a)Thermal stability of Poly (S-SA-DVB 1:1:3); b)Thermal stability of Poly (S-SA-DVB 1:1:5)

Regarding Se-based small fragments, we conducted supplementary thermal stability tests on the Poly(S-SA-DVB 1:1:3) and Poly(S-SA-DVB 1:1:5). These tests indicate that the selenium-containing components in the product remain stable up to the reaction temperature range (100–120 °C). Decomposition or elimination of Se-based fragments is unlikely under these conditions.


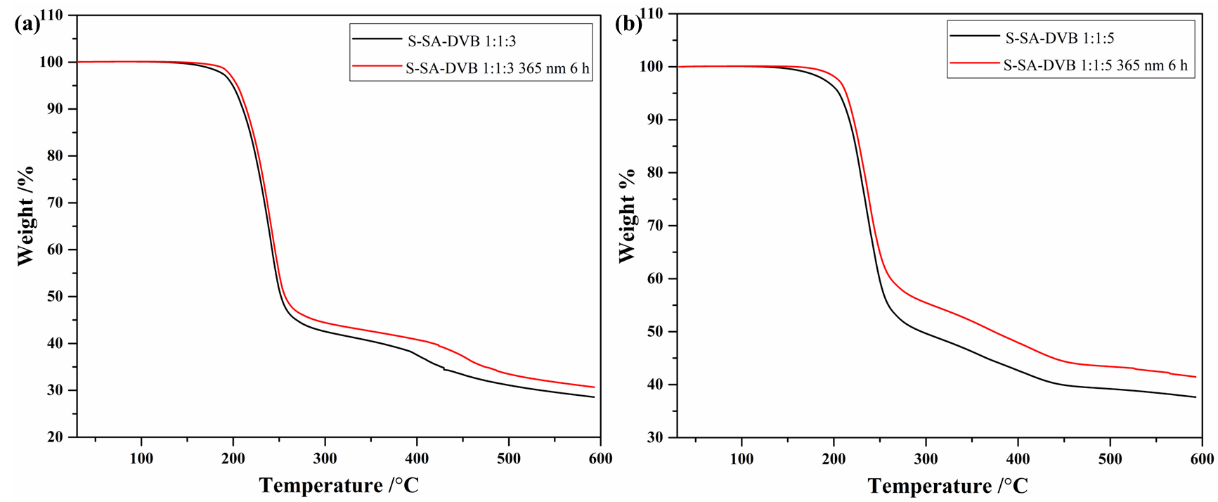


**Figure.S1****9** a) Comparison of thermal stability of Poly (S-SA-DVB 1:1:3) before and after 10 w 365 nm illumination; b) Comparison of thermal stability of Poly (S-SA-DVB 1:1:5) before and after 10 w 365 nm illumination

**Adhesion performance test：**


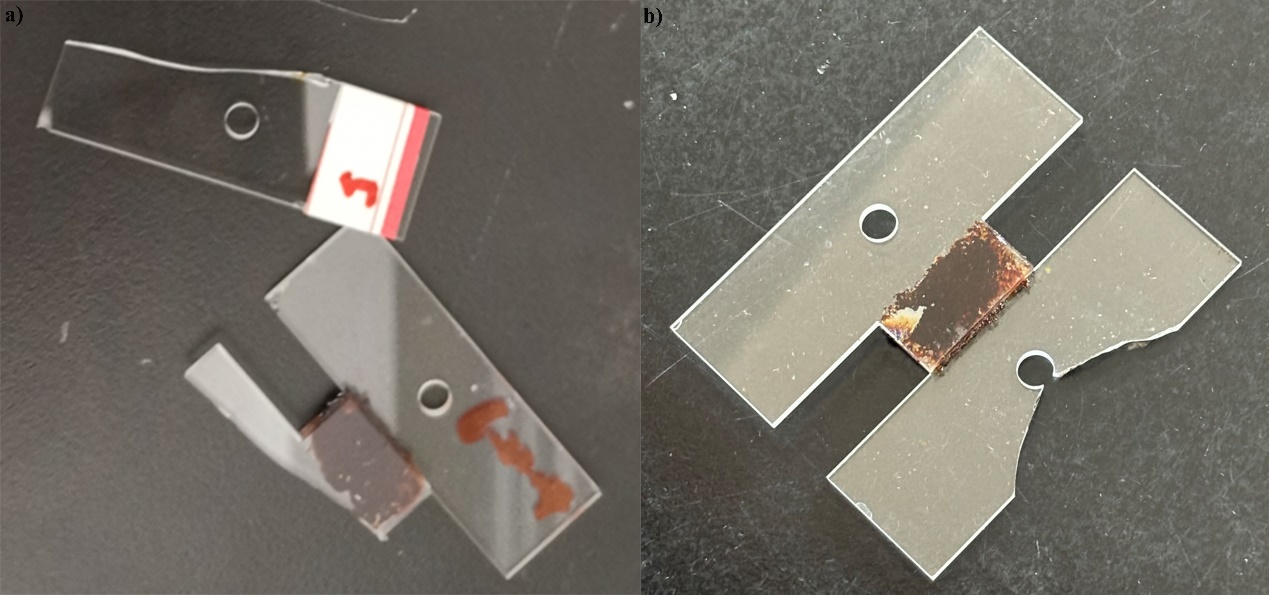


**Figure.S20** Broken glass substrate

**Adhesion performance test：**


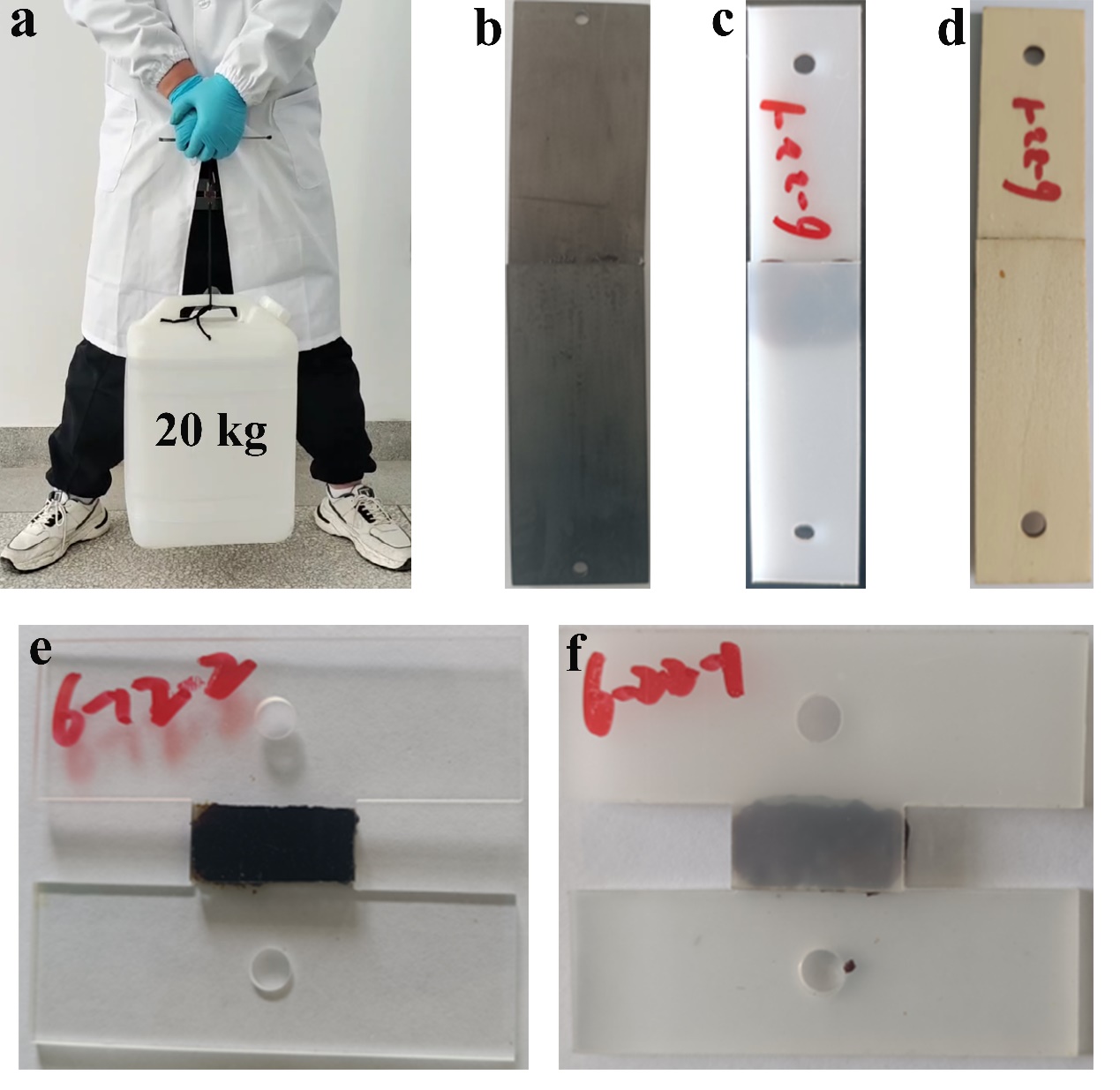


**Figure.S21**. Test the adhesion performance of different substrates using Poly(S-SA-DVB 3-4-1) as the adhesive. a) Actual diagram of adhesive performance testing using steel plates as the substrate; b) Steel plate; c) PVC; d) Wood; e) Glass; f) Alec plate

[1] F.Xu，Z.Yang，X,Chen, *Applied Chemical Industry* **2013**, *42*.763-767
